# Levothyroxine therapy reduces endocan and total cholesterol concentrations in patients with subclinical hypothyroidism

**DOI:** 10.11613/BM.2025.010703

**Published:** 2024-12-15

**Authors:** Tihana Serdar Hiršl, Koraljka Đurić, Marina Čeprnja, Ivana Zec, Marijana Kraljević Šmalcelj, Tomislav Jukić, Tanja Bobetić-Vranić, Anita Somborac-Bačura

**Affiliations:** 1 Synlab Croatia, Polyclinic for Medical Laboratory Diagnostics, Zagreb, Croatia; 2 Srebrnjak Children's Hospital, Zagreb, Croatia; 3 Croatia Polyclinic, Zagreb, Croatia; 4 Department of Clinical Chemistry, Sestre Milosrdnice University Hospital Centre, Zagreb, Croatia; 5 Department of Oncology and Nuclear Medicine, Sestre Milosrdnice University Hospital Centre, Zagreb, Croatia; 6 School of Medicine, University of Zagreb, Croatia; 7 Department of Medical Biochemistry and Hematology, Faculty of Pharmacy and Biochemistry, University of Zagreb, Zagreb, Croatia

**Keywords:** asymmetric dimethylarginine, endocan, endothelin-1, hypothyroidism, thyroxine

## Abstract

**Introduction:**

Subclinical hypothyroidism (SCH) is an independent risk factor for cardiovascular diseases due to endothelial dysfunction and atherosclerosis development. The aim of this study was to determine whether the levothyroxine therapy could impact the concentrations of endothelial dysfunction blood markers, namely endothelin-1 (ET-1), asymmetric dimethylarginine (ADMA) and endocan, in patients with a mild form of SCH (thyroid-stimulating hormone (TSH) ≤ 10 mIU/L).

**Materials and methods:**

In this case-control prospective study, SCH patients and healthy controls were recruited during their regular health examinations. Medical specialists prescribed levothyroxine to SCH patients if necessary. The endothelial dysfunction markers, as well as other biochemical markers, were measured in all subjects at baseline, and after 6 months of levothyroxine treatment following the euthyroidism.

**Results:**

Our study showed higher ADMA (248.00 (168.78-540.20) *vs*. 166.30 (140.60-243.40) μg/L, P = 0.002), endocan (114.30 (63.45-194.65) *vs*. 67.26 (50.80-126.10) ng/L, P = 0.004), low-density lipoprotein cholesterol (LDL) (3.3 ± 0.6 *vs*. 3.7 ± 0.9 mmol/L, P = 0.043) and non-high-density lipoprotein cholesterol (non-HDL) (3.8 ± 0.7 *vs*. 4.2 ± 1.0 mmol/L, P = 0.020) concentrations in patients with a mild form of SCH in comparison with healthy subjects. In SCH patients, after 6 months of levothyroxine treatment following the euthyroidism, we observed a significant decrease in endocan (91.47 (61.88-200.03) *vs*. 97.90 (55.18-154.88) ng/L, P = 0.004), and total cholesterol concentrations (CHOL) (6.2 ± 1.0 *vs*. 5.8 ± 1.0 mmol/L, P = 0.039).

**Conclusions:**

A mild form of SCH is associated with higher concentrations of endocan, ADMA, LDL and non-HDL. The potential benefits of levothyroxine therapy were shown through the significant decrease of endocan and CHOL concentrations in SCH patients, thus contributing the atherosclerosis prevention.

## Graphical abstract

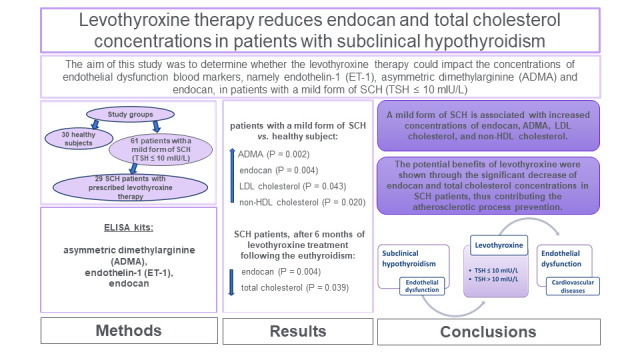

## Highlights

• Patients with SCH (thyroid-stimulating hormone (TSH) ≤ 10 mIU/L) showed higher concentrations of asymmetric dimethylarginine and endocan

• After 6 months of levothyroxine treatment following the euthyroidism, SCH patients showed a significant decrease in endocan and total cholesterol concentration

• Potential benefits of levothyroxine therapy were shown for patients with a mild form of SCH

## Introduction

Subclinical hypothyroidism (SCH) is a state characterized by higher serum concentrations of thyroid-stimulating hormone (TSH) and normal serum concentrations of free thyroxine (fT4) and free triiodothyronine (fT3) (*1*). Depending on the increased TSH concentrations in serum, SCH can be divided into mild (TSH ≤ 10.0 mIU/L) and severe (TSH > 10.0 mIU/L) forms of the disease. A mild form occurs in 75% of all patients with SCH (*2*). In general, SCH affects 4-10% of the adult population, it is more common in women, and the prevalence increases with age (*1*).

Although most SCH patients do not exhibit clinical symptoms, SCH can exert a damaging effect on cardiovascular system (*3*). Subclinical hypothyroidism is an independent risk factor for the development of atherosclerosis, congestive heart failure, coronary heart disease, ischemic heart disease, and consequent death (*4*-*7*). The association between SCH and cardiovascular diseases (CVD) was established, but the underlying molecular mechanism needs to be elucidated (*3*-*5*). It is assumed that this mechanism includes specific molecular pathways in endothelial cells causing nitric oxide (NO) decrease and other molecular changes, characteristic of endothelial dysfunction, thus resulting in the development of atherosclerosis and increasing the risk of CVD occurrence (*8*). Since SCH is characterized only by increased TSH concentration, the possible mechanism underlying is that the increase of TSH concentration can bind extra TSH receptors (TSHR) expressed on endothelial cells, thus resulting in a decrease of endothelial nitric oxide synthase (eNOS) and prostacyclin (PIG2) expression (*9*, *10*). It is assumed that SCH accelerates endothelial dysfunction through four aspects: dyslipidemia, chronic inflammation, oxidative stress, and insulin resistance, since all these factors are commonly found in SCH patients (*11*). Furthermore, thyroid hormone receptors are present on hematopoietic stem cells, so thyroid hormones may modulate the production of platelets and other blood cells leading to larger thrombocytes observed in SCH patients (*12*, *13*).

Moreover, SCH management is a common topic of debate. In clinical practice, levothyroxine is a prescribed medication for patients with TSH > 10.0 mIU/L. For patients with TSH ≤ 10.0 mIU/L, the introduction of levothyroxine therapy is individualized, depending on additional factors such as age, pregnancy planning and pregnancy, goiter, positive anti–thyroid peroxidase (anti-TPO) status, and presence of symptoms (fatigue, feelings of cold or dry skin, depression, heart function disorder, menstrual cycle disorder, *etc*.) (*14*). Therefore, the clinical importance and treatment of patients with a mild form of SCH are still controversial, and the treatment benefits are still insufficiently investigated and proven.

In this study, we hypothesized that patients with a mild form of SCH had significantly higher concentrations of endothelial dysfunction blood markers such as asymmetric dimethylarginine (ADMA), endothelin-1 (ET-1) and endocan (endothelial cell-specific molecule 1), as well as higher concentrations of high sensitivity C-reactive protein (hs-CRP), triglycerides (TG), total cholesterol (CHOL), low density lipoprotein cholesterol (LDL), non-high density lipoprotein cholesterol (non-HDL), and mean platelet volume (MPV) in comparison with the healthy subjects. Furthermore, we hypothesized that the introduction of levothyroxine therapy in patients with a mild form of SCH, the establishment of a euthyroid state, and continuation of the levothyroxine treatment for 6 months would have a beneficial effect on lowering these biomarkers. Therefore, the aim of this study was to determine whether the levothyroxine therapy could impact the concentrations of endothelial dysfunction (ADMA, ET-1, and endocan) and other biochemical markers in patients with a mild form of SCH.

## Materials and methods

### Subjects

In this case-control prospective study, participants were recruited during their regular health examinations in hospitals by medical specialists. Patients with SCH were recruited at the Department of Internal Medicine, Special Hospital Agram, and the Clinic of Oncology and Nuclear Medicine, Sestre milosrdnice University Hospital Centre, from September 2021 to January 2024. Based on the medical history, clinical examination and laboratory findings, the medical specialist who confirmed SCH diagnosis decided to which patient would be prescribed levothyroxine substitution therapy. Healthy individuals were recruited during the preventive annual check-up at the Department of Internal Medicine, Special Hospital Agram, during the same period. Sample analyses were performed in the Medical Biochemistry Laboratory, Special Hospital Agram, and in the Department of Clinical Chemistry, Sestre milosrdnice University Hospital Centre.

**Figure 1:**
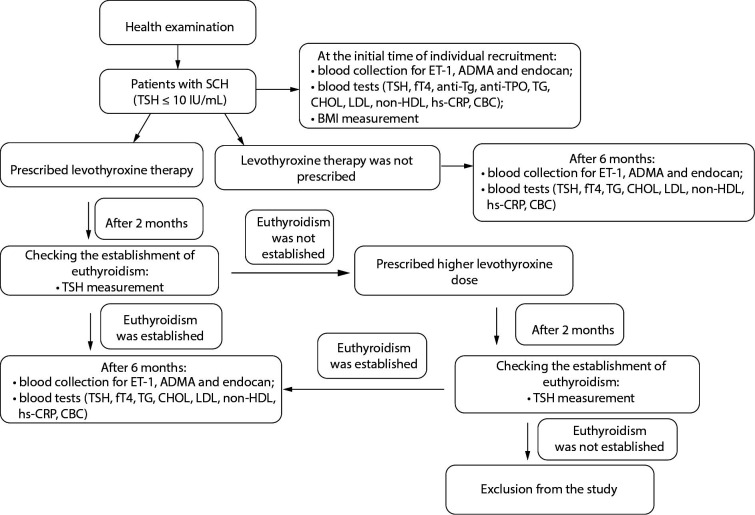
Flow chart of the study design. SCH - subclinical hypothyroidism. TSH - thyroid-stimulating hormone. fT4 - thyroxine. anti-Tg - anti-thyroglobulin. anti-TPO - anti-thyroid peroxidase. ET-1 - endothelin-1. ADMA - asymmetric dimethylarginine. TG - triglycerides. CHOL - cholesterol. LDL - low density lipoprotein cholesterol. non-HDL - non-high density lipoprotein cholesterol. BMI - body mass index. hs-CRP - high sensitivity C-reactive protein. CBC - complete blood count.

In our study, euthyroidism is defined as a concentration of TSH from 0.27-4.20 mIU/L, which is a reference range defined by the method and reagent manufacturer. For patients with prescribed levothyroxine replacement therapy, treatment usually started with 25 µg. The concentration of TSH was checked after 2 months, and the dose was increased if necessary. Biomarkers of interest were measured before the introduction of therapy and again 6 months after the established euthyroidism. A detailed flow chart of the study design is shown in the Figure 1.

The inclusion criteria for SCH patients were as follows: adults (18-75 years old), TSH concentration: 4.2-10.0 mIU/L, fT4 concentration within the normal range (11.9-21.6 pmol/L, as a reference range defined by the method and reagent manufacturer), and good general health as assessed by a complete medical and endocrine examination. The study included patients with SCH who were not under levothyroxine therapy at the time of recruitment. The inclusion criteria for the control group were the same as stated above, with an exception for the TSH concentration within the reference range.

The exclusion criteria for both SCH patients and controls were as follows: pregnancy, liver failure, chronic kidney failure, hypertension, respiratory failure, coronary heart disease, diabetes mellitus, malignancy, tobacco use, use of drugs that may affect the thyroid hormone concentration (amiodarone, steroids, lithium), use of statins, severe COVID-19 infection in the last two years, pituitary/hypothalamic disorders, systemic autoimmune diseases, and morbid obesity (body mass index (BMI) > 35 kg/m^2^).

Written informed consent was obtained from all participants, and the study was approved by ethical committees of Special Hospital Agram (No. 06-09-2021), Sestre milosrdnice University Hospital Centre (No. 251-29-11-21-11) and University of Zagreb Faculty of Pharmacy and Biochemistry (No. 251-62-03-21-34).

### Methods

Blood samples were obtained between 8:00 and 10:00 a.m. after a 12-hour fast. Blood samples were collected with ethylenediaminetetraacetic acid (EDTA) tubes (Greiner Bio-One, Kremsmünster, Austria) for complete blood count (CBC) (white blood cells (WBC), platelets (Plt), and mean platelet volume (MPV)). Serum samples were collected in tubes with a clot activator (Greiner Bio-One, Kremsmünster, Austria). After 30 minutes of spontaneous clotting, the samples were centrifuged at 2000xg for 10 minutes at room temperature. All the analyses were done on the same day as blood drawings, except for the serum ET-1, ADMA, and endocan concentrations. Serum for these three analyses were stored at - 80 ºC until the enzyme-linked immunosorbent assay (ELISA) measurements were performed.

Endothelin-1 (R&D Systems, Inc., Minneapolis, USA), ADMA (Elabscience, Houston, USA) and endocan (Abcam, Cambridge, United Kingdom) ELISA kits (for research use only) were used for serum measurements according to the manufacturers' instructions. The limit of detection (LOD) for ET-1, ADMA, and endocan were 0.031 ng/L, 9.38 μg/L, and 10.0 ng/L, respectively. Manufacturer analytical specifications for ET-1, ADMA and endocan are shown in Supplement 1.

Serum TSH, fT4, anti-TPO, and anti-Tg concentrations were measured on Cobas e601 autoanalyzer (Roche, Mannheim, Germany). The reference range for TSH and fT4 is defined as 2.5th-97.5th percentiles. According to the method and reagent manufacturer, cut-off values that indicate presence of autoimmune thyroid disorder for anti-TPO and anti-Tg are 34 kIU/L and 115 kIU/L, respectively, which is considered as positive results for antibodies. Triglycerides, CHOL and high density lipoprotein cholesterol (HDL) were measured on AU 480 autoanalyzer (Beckman Coulter, Brea, USA). LDL concentrations were determined using the Friedewald formula (LDL = CHOL – HDL – TG/2.2) or measured on AU 480 autoanalyzer (Beckman Coulter, Brea, USA) if triglycerides > 4.5 mmol/L. Non-HDL cholesterol was calculated using equation (non-HDL = CHOL – HDL). Hs-CRP concentrations were measured on AU 480 autoanalyzer (Beckman Coulter, Brea, USA). Leukocytes, Plt, and MPV were measured on DxH 800 autoanalyzer (Beckman Coulter, Brea, USA). For the common parameters, internal and external quality control assessments were carried out according to the laboratory-defined standard operating procedures throughout the whole study duration. Body mass index was calculated using the equation (body weight in kilograms divided by body height in meters squared).

### Statistical analysis

The Kolmogorov-Smirnov test was used to test the assumption of normal distribution. Depending on the normality, data were presented as mean and standard deviation or median and interquartile range. Age was presented as median and range. Accordingly, data were analyzed using the paired sample t-test or Wilcoxon rank sum test for paired samples and the independent sample t-test or Mann-Whitney test for independent samples. In the case of unclear results obtained by the Wilcoxon rank sum test for paired samples, the results were supplemented with the Hodges-Lehmann median difference analysis. P values less than 0.05 were considered statistically significant. Statistical data analysis was performed using the MedCalc statistical software version 20.013 (MedCalc, Ostend, Belgium).

## Results

Sixty-one adult patients with a mild form of SCH and 30 healthy individuals as controls (matched to the patient group for sex and age) were enrolled in this study. The demographic features and laboratory test results of the SCH patients and control subjects are presented in Table 1. As expected, TSH, anti-TPO and anti-Tg concentrations were significantly higher and fT4 concentration was lower in the SCH patients than in healthy controls. Both examined antibodies (anti-TPO and anti-Tg) were positive in 25 SCH patients, only anti-TPO was positive in 31 patients and only anti-Tg was positive in 29 patients. The results of endothelial dysfunction biomarkers showed that serum ADMA and endocan concentrations were significantly higher in the SCH patient group as compared to the control group but there were no differences in ET-1 concentrations.

**Table 1:** Clinical characteristics, endothelial dysfunction blood markers, baseline thyroid function test results and biochemical parameters of the control subjects and SCH patients

**Parameter (unit)**	**Controls (N = 30)**	**SCH patients (N = 61)**	**P**
Age (years)	43 (24-62)	46 (25-67)	0.161
Sex (female)	23	48	0.828
ET-1 (ng/L)	1.36 (1.09-1.56)	1.24 (1.07-1.46)	0.287
ADMA (μg/L)	166.30 (140.60-243.40)	248.00 (168.78-540.20)	0.002
Endocan (ng/L)	67.26 (50.80-126.10)	114.30 (63.45-194.65	0.004
TSH (mIU/L)	1.81 ± 0.82	7.19 ± 1.36	< 0.001
fT4 (pmol/L)	15.0 (14.0-16.0)	14.0 (13.0-15.0)	0.014
anti-Tg (kIU/L)	14.7 (14.2-15.2)	72.1 (15.7-209.4)	0.002
anti-TPO (kIU/L)	5.9 (5.0-7.9)	40.0 (7.3-131.2)	0.001
TG (mmol/L)	0.9 (0.7-1.3)	1.1 (0.8-1.5)	0.064
CHOL (mmol/L)	5.5 ± 0.9	5.9 ± 1.1	0.059
LDL (mmol/L)	3.3 ± 0.6	3.7 ± 0.9	0.043
non-HDL (mmol/L)	3.8 ± 0.7	4.2 ± 1.0	0.020
BMI (kg/m2)	23.9 ± 2.9	25.5 ± 3.9	0.046
hs-CRP (mg/L)	0.9 (0.5-1.2)	1.1 (0.6-1.9)	0.057
WBC (x10^9^/L)	5.5 (5.0-6.6)	6.2 (5.4-7.2)	0.132
Plt (x10^9/^/L)	263 ± 47	252 ± 45	0.285
MPV (fL)	8.5 (7.9-9.1)	8.3 (8.2-8.6)	0.726
Data are shown as mean ± standard deviation or as median (interquartile range). Age is presented as median and range. The difference between groups was tested by t-test or Mann-Whitney test. P < 0.05 was considered statistically significant. SCH - subclinical hypothyroidism. ET-1 - endothelin-1. ADMA - asymmetric dimethylarginine. TSH - thyroid-stimulating hormone. fT4 - thyroxine. anti-Tg - anti-thyroglobulin. anti-TPO - anti-thyroid peroxidase. TG – triglycerides. CHOL - cholesterol. LDL - low density lipoprotein cholesterol. non-HDL - non-high density lipoprotein cholesterol. BMI - body mass index. hs-CRP - high sensitivity C-reactive protein. WBC - white blood cells. Plt - platelets. MPV - mean platelet volume.			

Among the patients with confirmed SCH, 29 were prescribed levothyroxine substitution therapy, and 32 were without the need for it. The laboratory test results for the group of SCH patients on levothyroxine replacement therapy are presented in Table 2. Due to the levothyroxine therapy, serum TSH concentration returned within the normal range, but it was still significantly higher as compared to controls (P < 0.001). However, in three SCH patients TSH concentrations were above the reference range after 6 months of levothyroxine replacement therapy, although they achieved euthyroidism at the time of therapy introduction. On the other hand, the concentration of fT4 in SCH patients receiving the levothyroxine therapy reached the same concentration as in healthy subjects (P = 0.130). Among the endothelial dysfunction blood markers, the endocan concentration in SCH patients taking the levothyroxine therapy showed a significant decrease in serum (Hodges-Lehmann median difference of - 24.90, 95% confidence interval of - 52.20 to - 7.55, and P = 0.004), with values ​​no longer different from the control group (P = 0.180). However, treated SCH patients did not show significant changes in serum ADMA or ET-1 concentrations.

**Table 2:** Endothelial dysfunction blood markers, thyroid function test results and biochemical parameters of the SCH patients on levothyroxine therapy before (L0) and after 6 months of levothyroxine treatment following the established euthyroidism (L6)

**Parameter (unit)**	**SCH patients - L0** **(N = 29)**	**SCH patients - L6** **(N = 29)**	**P**
ET-1 (ng/L)	1.41 (1.23-1.52)	1.42 (1.22-1.61)	0.681
ADMA (μg/L)	332.60 (162.80-624.18)	353.20 (147.83-704.08)	0.456
Endocan (ng/L)	91.47 (61.88-200.03)	97.90 (55.18-154.88)	0.004
TSH (mIU/L)	7.60 ± 1.47	3.64 ± 1.70	< 0.001
fT4 (pmol/L)	13.0 (12.8-14.3)	17.0 (14.8-17.3)	< 0.001
TG (mmol/L)	1.1 (0.8-1.5)	1.1 (0.9-1.4)	0.864
CHOL (mmol/L)	6.2 ± 1.0	5.8 ± 1.0	0.039
LDL (mmol/L)	3.9 ± 0.8	3.6 ± 0.8	0.107
non-HDL (mmol/L)	4.5 ± 1.0	4.2 ± 0.9	0.144
hs-CRP (mg/L)	1.3 (0.7-3.0)	1.3 (0.8-2.5)	0.873
WBC (x10^9^/L)	6.1 (5.3-7.2)	5.5 (5.2-6.3)	0.049
Plt (10^9/^/L)	258 ± 47	267 ± 53	0.182
MPV (fL)	8.2 (7.6-9.1)	8.3 (7.6-9.7)	0.509
Data are shown as mean ± standard deviation or as median (interquartile range). The difference between groups was tested by the paired sample t-test or Wilcoxon rank sum. P < 0.05 was considered statistically significant. SCH - subclinical hypothyroidism. ET-1 - endothelin-1. ADMA - asymmetric dimethylarginine. TSH - thyroid-stimulating hormone. fT4 - thyroxine. TG – triglycerides. CHOL - cholesterol. LDL - low density lipoprotein cholesterol. non-HDL - non-high density lipoprotein cholesterol. hs-CRP - high sensitivity C-reactive protein. WBC - white blood cells. Plt - platelets. MPV - mean platelet volume.			

The laboratory test results for the group of SCH patients without levothyroxine therapy are presented in Table 3. Although these patients showed a significant decrease in TSH concentration after a follow-up of 6 months, it was still out of the normal reference range and significantly higher than in the controls (P < 0.001). On the other hand, after 6 months of follow-up, these patients showed an increased ET-1 concentration, while there were no significant changes in ADMA and endocan concentrations.

**Table 3:** Endothelial dysfunction blood markers, thyroid function test results and biochemical parameters of the SCH patients without levothyroxine therapy at the beginning of the study (NL0) and after 6 months of follow-up (NL6)

**Parameter (unit)**	**SCH patients - NL0** **(N = 32)**	**SCH patients - NL6** **(N = 32)**	**P**
ET-1 (ng/L)	1.15 (0.99-1.29)	1.25 (1.12-1.46)	0.003
ADMA (μg/L)	239.80 (190.95-431.90)	238.40 (160.45-449.05)	0.411
Endocan (ng/L)	133.70 (70.01-192.60)	126.95 (80.10-162.70)	0.970
TSH (mIU/L)	6.81 ± 1.16	5.43 ± 2.16	0.002
fT4 (pmol/L)	15.0 (13.7-17.0)	15.0 (13.0-16.0)	0.127
TG (mmol/L)	1.0 (0.8-1.5)	0.9 (0.7-1.1)	0.068
CHOL (mmol/L)	5.7 ± 1.1	5.5 ± 0.9	0.102
LDL (mmol/L)	3.4 ± 0.9	3.5 ± 0.7	0.635
non-HDL (mmol/L)	4.0 ± 1.03	3.9 ± 0.81	0.815
hs-CRP (mg/L)	0.9 (0.6-1.5)	1.0 (0.7-1.5)	0.652
WBC (x10^9^/L)	6.2 (5.5-7.3)	5.9 (5.0-7.3)	0.104
Plt (x10^9/^/L)	248 ± 43	243 ± 45	0.265
MPV (fL)	8.3 (8.0-9.0)	8.3 (7.9-9.0)	0.601
Data are shown as mean ± standard deviation or as median (interquartile range). The difference between groups was tested by the paired sample t-test or Wilcoxon rank sum. P < 0.05 was considered statistically significant. SCH - subclinical hypothyroidism. ET-1 - endothelin-1. ADMA - asymmetric dimethylarginine. TSH - thyroid-stimulating hormone. fT4 - thyroxine. TG - triglycerides. CHOL - cholesterol. LDL - low density lipoprotein cholesterol. non-HDL - non-high density lipoprotein cholesterol. hs-CRP - high sensitivity C-reactive protein. WBC - white blood cells. Plt - platelets. MPV - mean platelet volume.			

## Discussion

Our study showed higher endocan, ADMA, LDL and non-HDL concentrations in patients with a mild form of SCH (TSH ≤ 10.0 mIU/L) in comparison with the healthy subjects. In patients with a mild form of SCH, after 6 months of levothyroxine treatment and previously established euthyroidism, we observed a significant decrease in endocan and CHOL concentrations, while in SCH patients without levothyroxine therapy, we observed an increase in ET-1 concentration after a follow-up of 6 months.

To the best of our knowledge, this is the first study in this form that focused on the potential benefits of levothyroxine replacement therapy on blood markers of endothelial dysfunction in SCH patients with a mild form of the disease.

Previously, similar to our findings, Arpaci *et al*. found that serum ADMA and endocan concentrations were higher in SCH patients as compared to euthyroid subjects, and they also reported higher transforming growth factor beta (TGF-β) and hs-CRP in SCH patients (*15*). The other two studies showed significantly higher values of ADMA in SCH patients, and additionally Kumar *et al.* concluded that ADMA, as a NO synthase inhibitor, should be considered in the diagnosis of CVD in SCH patients (*16*, *17*). Gao *et al*. showed no significant differences in serum ET-1 and visfatin between SCH patients (TSH > 10.0 mIU/L) and controls, as well as between SCH patients (TSH < 10.0 mIU/L) and controls. In their study, only SCH patients with TSH > 10.0 mIU/L received levothyroxine therapy and after 6 months of treatment period there were no significant differences in serum ET-1 and visfatin. However, these two SCH groups showed significantly lower NO and omentin-1 basal serum concentrations, and a significant increase of these markers after 6 months of euthyroidism, indicating that reduced NO secretion plays a key role in endothelial dysfunction in SCH patients, while decreased adipokine omentin-1 contributes to endothelial dysfunction (*4*). In the study of Tilly *et al.*, ET-1 plasma concentrations in patients with thyroid gland disorders were significantly higher in patients with Hashimoto’s thyroiditis and Grave’s disease, but not significantly different in patients with endemic goiter as compared to healthy controls and patients with Graves’ disease (*18*). Another study showed that SCH in patients with metabolic syndrome significantly contributes to lowering the NO concentrations and ET-1 elevation (*19*). Although higher ET-1 concentrations appear to be frequent in autoimmune and inflammatory diseases because immune complexes stimulate endothelial cells to release ET-1, results may vary between studies investigating subclinical hypothyroidism (*20*). It is possible that no significant differences in ET-1 concentrations between patients with a mild form of SCH and healthy controls in our study were due to the low concentrations of ET-1 measured and its short half-life.

In this study, we also showed that patients with a mild form of SCH had a significantly higher LDL and non-HDL concentration than the control group. Geng *et al.* showed the association between higher concentrations of TSH and atherogenic lipid profile (higher TG and LDL) even in a mild TSH increase, especially in postmenopausal women (*21*). The other two studies showed significantly higher CHOL and LDL concentrations in SCH patients, together with higher hs-CRP and IL-6 (*16*, *17*). Another clinical study showed an increase in IL-6, tumor necrosis factor alpha (TNF-α), and CRP concentrations in SCH patients, as well as the correlation of the mentioned inflammatory markers with the flow-mediated dilatation parameter of the brachial artery (*8*). Though we did not find significant evidence of inflammation in the SCH patients, as well as no ET-1 increase, endothelial dysfunction was proven through increased ADMA and endocan concentrations. Since our study refers to a mild form of SCH, markers of inflammatory processes such as hs-CRP and leukocytes might be insufficiently sensitive. In patients with SCH, changes in the values of the platelet index MPV were also observed, although conflicting results have been reported. One study showed no changes in platelet count, but MPV was significantly higher (*13*). In our study, there were no differences in either platelet count or MPV index between the patients with a mild form of SCH and the control group, which is in agreement with another study that showed no changes in MPV and hs-CRP in SCH patients (*22*).

As our study showed, managing the TSH concentrations in patients taking substitution therapy can be challenging, and other studies also suggest that it is not uncommon for participants in clinical studies to remain outside of the therapeutic target range at the end of the trial. Furthermore, the ideal TSH values for patients receiving levothyroxine therapy remain uncertain (*23*). Ozcan *et al.* focused on patients after one month of stable euthyroidism and reported a significant decrease in ADMA and hs-CRP concentrations in blood, which is opposite to no significant changes in serum ADMA and hs-CRP values in our SCH patients on levothyroxine therapy (*17*). In our study, there was also no significant reduction in serum TG despite months of therapy. This may be the reason for the absence of ADMA decrease due to the mechanism by which hyperlipidemia interferes with NO synthesis and increases the ADMA concentration. On the other hand, endocan as a novel biomarker for the evaluation of endothelial dysfunction, significantly decreased in SCH patients on levothyroxine therapy. A recent review pointed out endocan, due to its high specificity and reproducibility reflecting endothelial function, disease state, and improving cardiovascular risk stratification (*24*). In our study, patients on levothyroxine therapy showed a significant decrease in CHOL concentration. We found this very important because our patients in the SCH group without therapy did not show any significant changes in their lipidemic state. The efficacy of replacement therapy on lipid parameters showed discrepancies and conflicting results in previous studies. In one study, after a month of stable euthyroidism, they found that levothyroxine therapy replacement did not show an effect on TG, CHOL, or LDL concentrations, and in another study the same was shown after three months of euthyroidism (*17*, *25*). Contrary to this, Monzani *et al.* found a significant decrease in serum CHOL and LDL concentrations after 6 months of euthyroidism (*26*).

Lastly, a significant increase in ET-1 concentration after 6 months of follow-up that we found in SCH patients without levothyroxine therapy is probably due to low concentrations of measured ET-1 that turned out to be just statistically significant, and not clinically.

The main limitation of our study could be the small sample size, which makes it difficult to obtain clear conclusions about the benefits of levothyroxine treatment in patients with a mild form of SCH. More studies with larger patient populations are needed to elucidate this problem more completely.

In conclusion, our study showed that patients with a mild form of SCH had higher concentrations of cardiovascular disease risk factors, namely endocan, ADMA, LDL, and non-HDL. The potential benefits of levothyroxine therapy were shown through the significant decrease of endocan and CHOL concentrations in SCH patients, thus contributing the atherosclerosis prevention.

## Data Availability

The data generated and analyzed in the presented study are available from the corresponding author on request.
